# The Field of Science

**Published:** 1894-04-28

**Authors:** 


### The Field of Science.

The Liverpool Science and Arts Committee have received an anonymous offer of £5,000 towards the cost of erecting central buildings for the School of Science, Technology, and Arts, on condition that the Corporation subscribe a like amount.

It says all that can well be said of the quality of the water supply of Paris when we find a suggestion being made to the Académie of Medicine that that august body should take upon themselves to officially counsel all good citizens against drinking water in an unfiltered form. Doubtless M. Bucquoz, who made the suggestion, knows well what he is about; but surely even the most unwary among us knows now of what avail is the so-called filtering of water, when such is declared to contain 15,000 microbes to the cubic centimetre! For such a liquid (solid it might almost be called), boiling would be the least stringent measure worthy of the name. Typhoid fever abounds still, as it has done for some time, in the French capital; and who, indeed, can be surprised? The awakening of Paris to the dangers of insanitation has surely yet to come.

The recent agitation in France concerning the reform in medical education and examinations turned principally on the question whether the study of classical language should be included in the curriculum. A commission, comprising the Rector of the Faculty of Medicine and five Professors, returned their answer to the following intent: The physician is obliged to use a terminology which is derived from the Greek and the Latin. Although he may—without having been instructed in the classics—in the course of time acquire a superficial knowledge of the expressions, still there will remain in such a case a sentiment of inferiority, because he does not know their origin. In the interest of the profession the sentiment should be spared the future physician. Though there is a certain indirectness in this reply, there can be but one reading of the decision. In addition to the above, the commission wisely suggested the expediency of the medical faculty being conversant with at least one modern language. Indeed, as a contemporary points out on this subject, considering the present condition of medical science, deriving as it does its elements from all parts of the world, every physician should be somewhat of a polyglot.

It is perplexing to notice the increase of insanity in certain American States. The annual report of the New York State Board of Charities, submitted to the Legislature last month, shows a total increase in the number of insane in the institution of the State, from October 1st, 1880, to October 1st, 1893, of 8,843. Thus, whilst the increase in the population from 1880 to 1892 was 28 per cent., the increase of the insane in the institution was 83 per cent.

According to Volland, who writes in the *Zeitschrift für Klinische Medecin*, there are other than air-borne theories accountable in the pathology of phthisis. In his paper we find Volland substituting for this particular theory his belief that the bacilli are conveyed mechanically from the floor to the nasal and buccal mucous membranes. In early childhood this, he points out, is especially the case, when infants crawl about and play on the ground, learning to walk from touching the floor with their hands. The microbes thus find their way through the lymph glands of the neck to the apices of the lungs. Further, Dr. Volland enlarges on the frequency of strumous glandular disease in early life, which, in his opinion, more fully substantiates his theory. For this reason, the nasal mucous should, he further suggests, be constantly removed.

Of all the plants used for food there is none which has been so long known or has had, so to speak, more distinguished a lineage than asparagus. An article in a current monthly draws our attention to the record of this vegetable, which reaches back almost to the commencement of authentic history. Cratenus, the comic poet, mentioned it 425 years B.C. Some centuries later on, Pliny, in his "Natural History," about 60 A.D., has more to say on the subject. "Of all the productions of your gardens," he feelingly observes, "your chief care will be your asparagus," to which subject the writer devotes several chapters. He asserts that even in his day the soil about Ravenna was so favourable to the production of this vegetable that three heads grown in that district had been known to weigh a Roman pound. "Asparagus" observes our later authority, "being a Southern plant, the original stock found in Italy was of a more vigorous growth than that of more northern climes. It occurs all round the shores of the Mediterranean. In its wild state it is never found with us away from the sea coast."

